# Quantitative Spatial and Temporal Analysis of Fluorescein Angiography Dynamics in the Eye

**DOI:** 10.1371/journal.pone.0111330

**Published:** 2014-11-03

**Authors:** Flora Hui, Christine T. O. Nguyen, Phillip A. Bedggood, Zheng He, Rebecca L. Fish, Rachel Gurrell, Algis J. Vingrys, Bang V. Bui

**Affiliations:** 1 Department of Optometry and Vision Sciences, The University of Melbourne, Parkville, Victoria, Australia; 2 Neusentis, Pfizer Research and Development, Grant Park Science Park, Cambridge, United Kingdom; University of Florida, United States of America

## Abstract

**Purpose:**

We describe a novel approach to analyze fluorescein angiography to investigate fluorescein flow dynamics in the rat posterior retina as well as identify abnormal areas following laser photocoagulation.

**Methods:**

Experiments were undertaken in adult Long Evans rats. Using a rodent retinal camera, videos were acquired at 30 frames per second for 30 seconds following intravenous introduction of sodium fluorescein in a group of control animals (n = 14). Videos were image registered and analyzed using principle components analysis across all pixels in the field. This returns fluorescence intensity profiles from which, the half-rise (time to 50% brightness), half-fall (time for 50% decay) back to an offset (plateau level of fluorescence). We applied this analysis to video fluorescein angiography data collected 30 minutes following laser photocoagulation in a separate group of rats (n = 7).

**Results:**

Pixel-by-pixel analysis of video angiography clearly delineates differences in the temporal profiles of arteries, veins and capillaries in the posterior retina. We find no difference in half-rise, half-fall or offset amongst the four quadrants (inferior, nasal, superior, temporal). We also found little difference with eccentricity. By expressing the parameters at each pixel as a function of the number of standard deviation from the average of the entire field, we could clearly identify the spatial extent of the laser injury.

**Conclusions:**

This simple registration and analysis provides a way to monitor the size of vascular injury, to highlight areas of subtle vascular leakage and to quantify vascular dynamics not possible using current fluorescein angiography approaches. This can be applied in both laboratory and clinical settings for *in vivo* dynamic fluorescent imaging of vasculature.

## Introduction

The retina is the only place in the body that allows non-invasive, direct *in vivo* visualization of neuronal tissue and vasculature. Retinal imaging is an important laboratory and clinical tool for the study of ophthalmic disease [1] and increasingly retinal blood vessel changes are shown to help stratify the severity of systemic disease [2]. Retinal imaging can be undertaken with or without the aid of a contrast agent. The addition of a contrast agent such as sodium fluorescein provides additional information regarding the integrity of the retinal vasculature. Since the early 1900s intravenous injection of sodium fluorescein has been shown to temporarily highlight retinal vessels [3], [4], thus improving *in vivo* assessment of the retinal circulation and blood-retinal-barrier integrity [5]–[7]. It has since been adopted as a clinical tool for a host of conditions including glaucoma, diabetes, vascular occlusion, neovascularization and inflammatory disease [4], [8]–[10]. In particular, the recent development of ultra wide-field fluorescein angiography has allowed for easier detection of peripheral blood-retinal-barrier disruption [8], [11], [12]. Pre-clinically, fluorescein angiography has also been utilized in studies of vascular disease, angiogenesis and blood-neural-barrier compromise [13]–[16].

The standard approach to fluorescein angiography is to take a series of photographs at various times (several seconds to minutes) following fluorescein delivery to demonstrate choroidal, arterial and venous filling and clearance. These images are used to identify areas of vascular non-perfusion or retained fluorescence, which may highlight vascular compromise as indicated by fluorescein leakage. The intermittent recording and lack of accurate timing however, limits the analysis of fluorescein images to a qualitative assessment, almost a binary outcome. Thus the sensitivity to detect subtle changes, including differentiating vascular leakage from angiogenesis, is hampered. Video angiography provides the potential for quantitative analysis of more subtle differences in filling and leakage that may occur with vascular changes in disease [17]–[19]. To date, most studies that analyze video angiography have only examined the time it takes for fluorescein to appear in particular regions of interest [20]–[22]. Whilst this is useful, it does not provide insight to the degree and extent to which the vasculature in any given location may be altered. A method was developed by Hipwell et al [23], to analyze the entire image in human fluorescein angiograms. Although the analysis was limited to identifying the time to reach maximal fluorescence, it demonstrated the potential that analysis of the whole image has in assessing retinal vascular disease.

Here we describe a novel means to quantitatively measure fluorescein angiography profiles. Combining systematic fluorescein delivery, video angiography and a precise analysis, the spatial and temporal characteristics of rodent fluorescein angiography dynamics can be quantified. To test the robustness of the analysis method, we used the approach to identify areas of altered fluorescein angiography dynamics following acute focal laser photocoagulation injury on a separate cohort of rodents.

## Materials and Methods

All experimental methods and animal care procedures conformed to the Association of Research in Vision and Ophthalmology, the National Health and Medical Research Council of Australia's guidelines for animal care and experimentation [24] and was approved by the University of Melbourne Animal Ethics Committee (1111991.1).

### General animal preparation

Experiments were performed on adult, *Long-Evans* rats (n = 14, 336±11 g) housed in a temperature and light controlled (20°C, <50 lux, 12 hour light/dark cycle on at 8 am) animal facility. Normal rat chow (AIN93G) and water were available *ad libitum*.

General anesthesia was induced with an intramuscular injection of ketamine and xylazine (60 mg/kg and 5 mg/kg respectively, Troy Laboratories Pty Ltd, Smithfield, NSW, Australia) prior to surgery and imaging. Corneal anesthesia and mydriasis were achieved with topical proxymetacaine (0.5% Alcaine, Alcon Laboratories, Frenchs Forest, NSW, Australia) and tropicamide (0.5% Mydriacyl, Alcon Laboratories) respectively. A custom-made water heating pad and immersion heater ensured body temperature was maintained at 37°C (Tempette Junior TE-8J, Techne, New Jersey, USA).

### Fluorescein angiography and blood pressure monitoring

**Figure 1:**
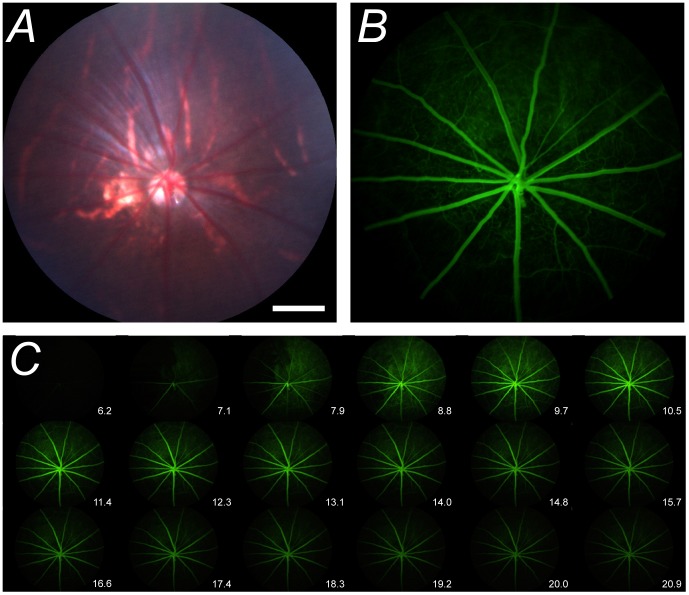
Video fluorescein angiography in rat retina. **A–B**. Representative bright-field image and its corresponding fluorescein image 10 seconds post fluorescein administration. **C**. Montage demonstrating the fluorescein transit through the retinal blood vessels for times between 6.2 and 20.9 seconds after the start of fluorescein infusion. Montage shows every 20^th^ frame taken from **Video S1**. White bar in Panel A represents 500 µm.

For intravenous delivery of sodium fluorescein, a femoral vein cannulation was performed using polyethylene tubing (inner diameter 0.28 mm, outer diameter 0.61 mm, Microtube Extrusions, NSW, Australia). Blood pressure was continuously monitored with a Powerlab system (ML110G, ADInstruments, NSW, Australia) and LabChart software (ADInstruments, Australia) via a femoral artery cannula. A bolus of sodium fluorescein (1%, 100 µl/kg) was intravenously delivered at 1.05 ml/min for 4 seconds with a syringe pump (Harvard Standard Pump 22, Serial #28974, Harvard Apparatus, MA, USA). Images of the posterior pole were taken from the start of fluorescein infusion with the Micron III rodent fundus camera (Phoenix Research Labs, CA, USA) with a 435–469 nm excitation filter and 520–530 nm barrier filter at 30 frames/sec using Streampix software (NorPix Inc., Quebec, Canada) for 30 seconds. Data were converted into tiff image stacks for offline analysis (Figure 1).

### Laser-induced blood-retinal-barrier disruption

Retinal tissue injury was induced by Nd: YAG focal laser photocoagulation (532 nm, 1.34 mW.s, 0.5 s, 100 µm diameter) in four sectors of the retina to produce disruption of the blood-retinal-barrier. This laser power was insufficient to disrupt Bruch's membrane, the layer separating the retinal pigment epithelium from the choroid, as no bubbling of the tissue or hemorrhaging was evident. This was confirmed using optical coherence tomography (Micron III, Phoenix Research Labs). Imaging was undertaken 30 minutes post-injury with fluorescein angiography. Post-laser imaging was extended to 60 seconds to capture the fluorescein leakage to greater extent. It is worth noting that whilst the use of laser photocoagulation to induce extravascular leakage mainly affects areas between the large blood vessels, the analysis method can also be applied to changes in fluorescein dynamics occurring within the arteries and veins.

### Fluorescence profile analysis methods

#### Image registration

All images were registered using custom written MATLAB scripts (R2013a, The MathWorks Inc., Massachusetts, USA; see Files S1–S8) via cross-correlation to a reference image, chosen to be when all vessels were filled with fluorescein (∼15 seconds from the start of fluorescein infusion). A simple x/y translation correction was sufficient to eliminate most image movement associated with animal breathing and small eye movements. Rigid and affine registration approaches provided little additional advantage. Minor contributions from rotation, scale and shear remained at times, however we found that attempting to correct these often produce more variability and significantly extended computation time. Instead, it was more practical to ameliorate small residual motion artifacts by PCA motion artifacts were ameliorated by PCA filtering as described below.

#### Principal component analysis (PCA) filtering

**Figure 2:**
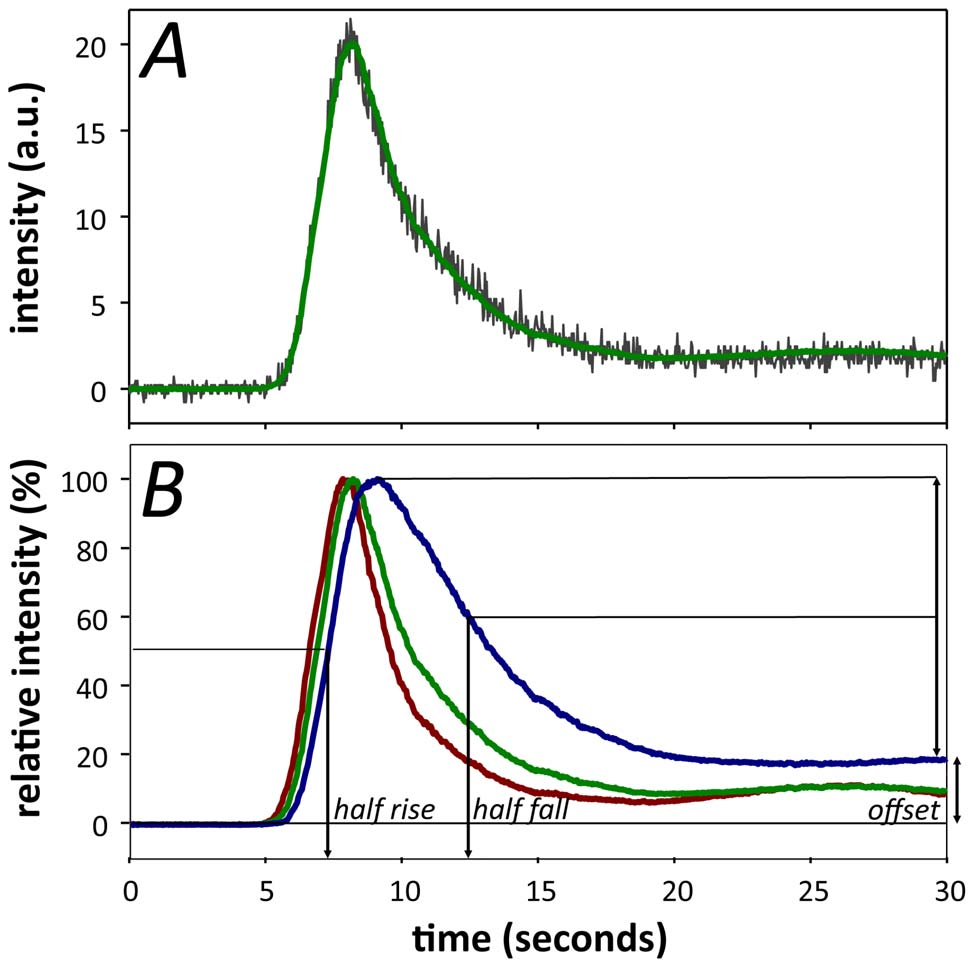
Video fluorescein dynamics in rat retinal vessels. **A**. Representative intensity profile for an individual pixel before (grey trace) and after PCA (green trace). **B**. Representative normalized fluorescein intensity profile as a function of time in an artery (red), vein (blue) and capillary/choroid (green). Parameters used to analyze fluorescence profiles include the half-rise, half-fall and offset amplitude.

Custom software written in MATLAB (see Files S1–S8) was used to quantitatively assess intensity profiles for each pixel. Pilot studies showed that a 30 second acquisition epoch fully captured the fluorescence transit in normal eyes, with the profile showing asymptotic behavior by 25 seconds (see Figure 2). Following image registration, PCA was used to smooth the data and remove artifacts associated with any remnant movement that was not corrected by translational image registration. In brief, PCA is a mathematical procedure of pattern recognition. It takes a data set made up of observations (in this case, image pixels) that can be described by several dependent variables (in this case, image frames) and represents the data by a new set of orthogonal variables called principal components [25], [26]. The first component accounts for the maximal amount of variance in the data, which for our usage is equivalent to finding the shape of the intensity profile that best fits the greatest number of pixels analyzed. If this component is removed, the second principal component explains the maximal remaining variance, and so forth. PCA extracts the most important information from the data and thus simplifies the data set. Two principal components were found to be sufficient to explain at least 95% of the variance in each of our image sequences in the control cohort, rendering the smoothed curves shown in Figure 2.

#### Extracted intensity profile parameters

**Figure 3:**
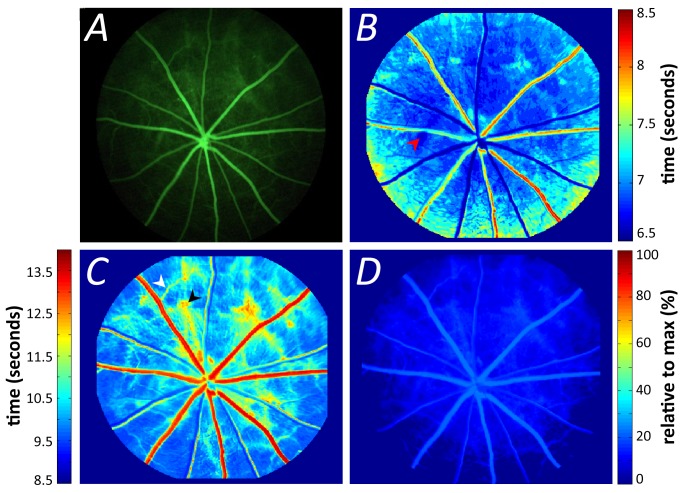
Pixel-by-pixel analysis of retinal video fluorescein angiography. (**A**). The half-rise (**B**) and half-fall (**C**) parameters of the same retina. Warmer colors indicate increasing delay in the parameter. Arrowheads indicate areas of early choroidal filling (red), delayed fluorescein clearance due to secondary venules (white) and delayed choroidal clearance (black). The offset amplitude (**D**) expressed relative to maximum fluorescence (%).

**Figure 4:**
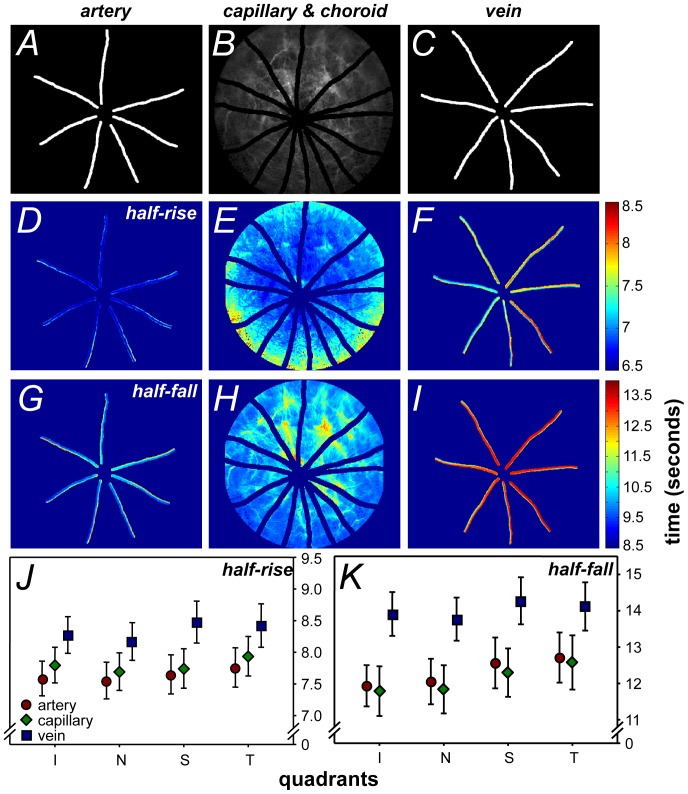
Segmentation of pixel-by-pixel analysis by blood vessel type. **A–C**. Development of the mask via manual vessel tracing: artery, capillary/choroid and vein respectively. **D–F**. Representative images of the half-rise of each pixel. **G–I**. Representative images of the half-fall of each pixel. **J–K**. Group average of the half-rise and fall respectively, across the four quadrants of the retina shows fluorescein filling in the order of arteries, capillaries and veins followed by their decay. Significant differences in rise (p = 0.01) and fall (p<0.001) were found between vessel types (two-way ANOVA). Data shown is mean ±SEM, n = 14.

Following PCA, the intensity profile at each pixel was determined. Parameters could then be extracted to describe the fluorescence dynamics including the half-rise, the time to reach 50% fluorescence; offset amplitude, the remnant fluorescence taken as an average of the last second of recording, expressed relative to the peak intensity; and the half-fall, the time to 50% decay between the peak and offset amplitude (see Figures 2, 3 and 4). Whilst PCA is excellent for imaged fields showing normal variance, it is less suited to fields with high variance such as those with focal laser injury. Thus for laser-injury data, a three-point moving median filter was applied to the data instead. Estimates of half-rise, half-fall and offset for the laser-injury data were then derived from the intensity profiles at each pixel.

#### Blood vessel tracing

**Figure 5:**
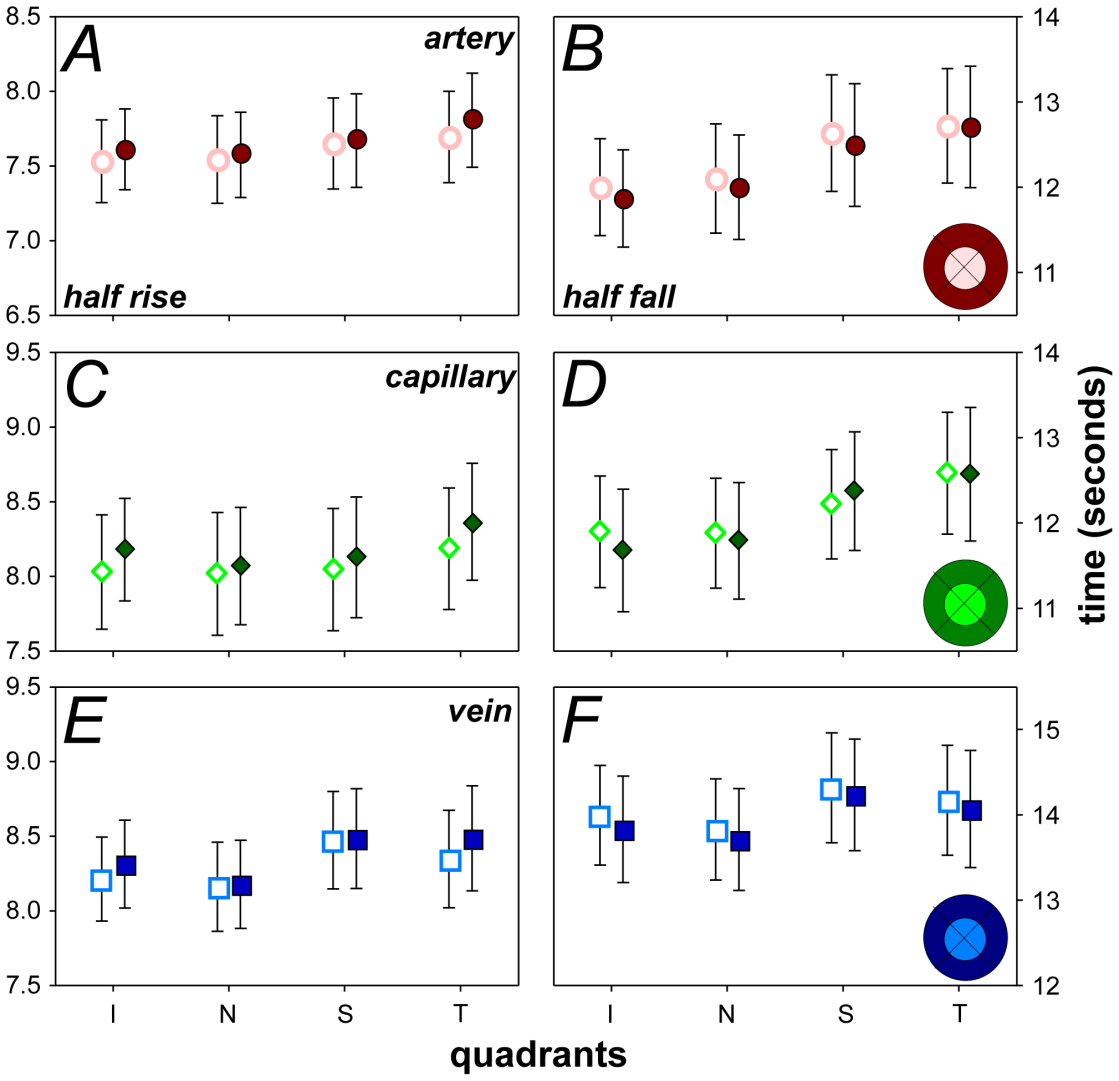
Half-rise and half-fall characteristics across two eccentricity zones. Group averaged comparison of changes to half-rise and half-fall time in arteries (**A–B**), capillaries (**C–D**) and veins (**E–F**). Inset shows how the retina was divided into 4 sectors with two eccentricity zones forming the inner (light shade) and outer zones (dark shade). Unfilled markers represent the inner zone and filled markers show the outer zone. Data shown is mean ±SEM, n = 14.

To analyze the blood vessel types separately, manual tracing was used to segment the major retinal arteries and veins from the rest of the fundus, which was taken to be the capillaries and choroid (see Figure 4B). Optic nerve fluorescence was masked from analysis. Whilst not employed in this study a robust automatic blood vessel segmentation algorithm would speed up analysis. However, automatic vessel segmentation should not impact the results of individual pixel data shown in this study. The retina was divided into four quadrants, corresponding to the superior, nasal, temporal and inferior retina. First, differences in fluorescein angiography dynamics between blood vessel types were considered. Second, to consider changes in dynamics that may occur with distance from the optic nerve head, the images were further divided into 2 zones by drawing two annuli: the first, inner zone with a 640 µm radius; the second, outer zone with a 1280 µm radius, centered on the optic nerve head (see Figure 5 inset).

#### Identification of abnormal pixels

**Figure 6:**
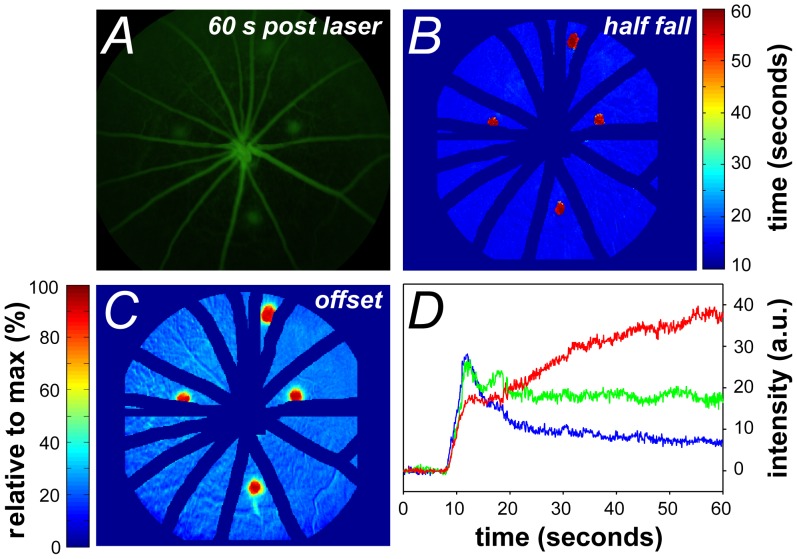
Video Fluorescein dynamics following laser injury. **A**. Fluorescein angiogram leakage 60 seconds post dye delivery. **B**. Pixel-by-pixel analysis highlights areas of prolonged half-fall in laser-injured retina. **C**. Analysis of offset amplitude highlights the extent of elevated offset post-laser injury. **D**. Representative intensity profiles of two pixels within a laser-burned region highlighting the diversity in fluorescence dynamics. The red trace demonstrates continuous increase in fluorescence; green trace shows minimal fluorescence decay plotted against a representative intensity profile in the control cohort (blue trace). Data expressed in raw intensity with arbitrary units, colours of trace correlate to the relative offset of the pixel.

**Figure 7:**
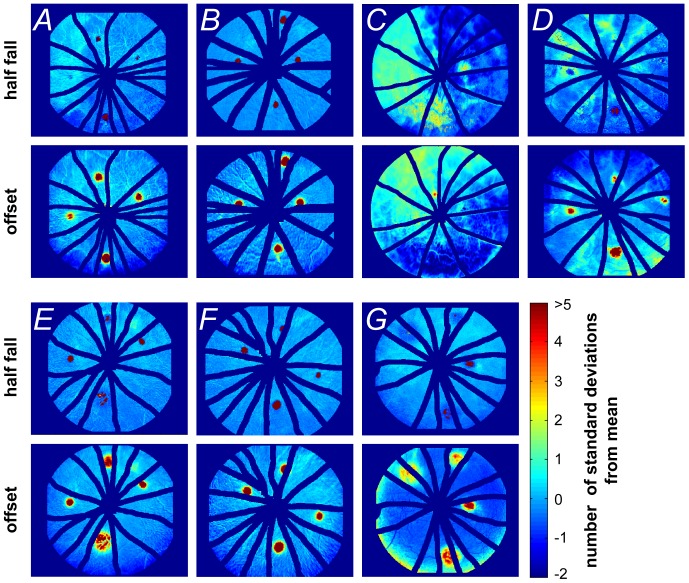
Half-fall and offset characteristics following laser injury in rat retina. Retinal probability maps identifying the areas of abnormal half-fall (1^st^ and 3^rd^ rows) and offset amplitude (2^nd^ and 4^th^ rows) in relation to the number of standard deviations from the mean of the whole image, n = 7 eyes. Panels A to G represent each of the 7 laser-injured retinas.

To identify abnormal pixels that fill or decay slower, one can compare against a baseline image sequence or a control cohort. Alternatively, quantification can be undertaken within the same image sequence, which has the advantage that no baseline sequence is needed and variability associated with measurements between sessions or between animals can be minimized. To achieve this, the half-fall and offset amplitude were used for comparisons. The half-fall and offset amplitude were chosen as we hypothesized that these parameters would be more affected by laser injury which can induce blood-retinal-barrier disruption and vascular leakage of fluorescein. It is worth noting that despite extending post-laser sequences out to 60 seconds, half-fall could not be identified in some pixels (see Figure 6D). In those cases, the half-fall time was recorded as 60 seconds. Similar to a Grubbs' test for outliers, Equation 1 was used to identify for each pixel the number of standard deviations away from the mean; where x =  half-fall or offset amplitude for a single pixel, µ =  average half-fall/offset for that eye, σ =  standard deviation for that eye. The number of standard deviations from the mean represents probabilities that a highlighted pixel randomly falls outside the normal range, which can be between 1 and 4 SD (see Figure 7).
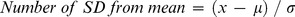
(1)


### Statistical analysis

All data are shown as group mean ±SEM. Parameters were compared using two-way ANOVA to assess for differences in the quadrants and eccentricity comparisons with Tukey's multiple comparisons test for post-hoc analysis. Statistical significance is indicated when p <0.05.

## Results

### Dynamic fluorescein angiography

Compared to *en face* fundus images taken under broad-spectrum light (Figure 1A), the presence of fluorescein acts as a contrast medium that improves the resolution of both major and minor retinal vessels (Figure 1B). Dynamic angiography provides clear delineation of the arterial and venous phases of fluorescein filling as well as their relative decay kinetics (please see Video S1). Figure 1C shows a montage of every 20^th^ frame over the course of the same fluorescein angiogram sequence.

### Pixel-by-pixel analysis reveals spatial profiles in the dynamics of fluorescein onset and offset

Figure 2A shows the difference in the intensity profiles for a representative pixel chosen in an area outside of the major retinal vessels, before and after PCA. The raw data (grey trace) contains inherent variability, which can be due to remnant image movement and background noise. By utilizing PCA, this noise can be reduced (green trace) without alteration of the underlying fluorescein angiography dynamics. Figure 2B shows a representative intensity profile taken from 3 individual pixels from an artery, vein and a capillary/choroidal area away from a major vessel. As the choroidal vasculature directly underlies the retinal vasculature, the capillary fluorescence profile also contains contributions from the choroid. This can be particularly evident in areas where there are breaks in the pigmentation of the retina.

Following fluorescein administration, the dye appears in the separate blood vessel types at a similar time (Figure 2B). Fluorescence then quickly reaches its peak intensity before decaying more slowly. After 25 seconds, fluorescence is relatively stable with the veins retaining greater fluorescence than the arteries and capillaries/choroid.

To explore fluorescein angiography dynamics of the posterior retina, the pixel-by-pixel analysis method was developed, allowing both spatial and temporal changes in retina blood vessels to be simultaneously analyzed (Figure 3). Figure 3B shows that the half-rise of the arteries occurs first but can be difficult to differentiate as their half rise times are only slightly faster (∼0.1 ms to 0.3 ms) than the surrounding areas of capillaries and choroid. This indicates that arteries and capillaries have similar half-rise times and that veins are slower by about 1 second in the rat, compared with arteries (warmer colors in Figure 3B). The differences between the various vessel types become more apparent with the half-fall as shown in Figure 3C. In particular the arteries show a rapid decay but the veins show a substantially slower decay to half brightness. It is also apparent that particular areas between the major vessels fill and decay more slowly (arrow heads in Figure 3C). We believe that this may arise due to the presence of secondary venules (white arrow head) and delayed choroidal filling (black arrow head) due to the watershed zones in the choroidal vascular bed as described by Hayreh [27]. After 30 seconds, fluorescence reaches asymptotic levels with the veins retaining greater fluorescence than the rest of the retina (Figure 3D).

To quantify differences between blood vessel types, large arteries and veins were segmented by manual tracing (Figure 4A–C) and analyzed separately from the rest of the fundus image. Figure 4D–F and 4G–I show the half-rise and half-fall, respectively in a representative animal. Figure 4J–K shows the comparison in half-rise and half-fall between blood vessel types in the four retinal quadrants. Significant differences were found between blood vessel types for half-rise (F_2,156_ = 5.79, p<0.01) and half-fall (F_2,156_ = 10.10, p<0.001) but not between quadrants. On average, veins showed a slower half-rise (8.3±0.1 s, 95% confidence limits: 6.1–10.6 s) compared to arteries (7.6±0.1 s, 5.5–9.8 s) and capillaries (7.8±0.1 s, 5.6–10.0 s). This is consistent with data reported in previous studies [28]–[30]. A greater difference in half-fall was apparent (Figure 4K), as veins were much slower to decay (13.9±0.3 s, 9.5–18.5 s) compared with arteries (11.9±0.3 s, 7.6–17.0 s) and capillaries (11.8±0.3 s, 7.2–17.1 s).

To consider changes to fluorescence dynamics with eccentricity, pixels were averaged in two eccentricity zones forming an inner (640 µm radius) and outer zone (1280 µm radius) as shown in the inset of Figure 5. No significant differences were found with eccentricity for the half-rise (Figure 5A,C,E) and half-fall (Figure 5B,D,F) for all vessel types. Smaller pixel bins were also used in a separate analysis; i.e. instead of 640 µm radius zones, 200 µm zones were used, but no significant change with eccentricity was apparent (data not shown). Differences in fluorescein dynamics may become apparent if we were able to image further into the periphery with the camera. By centering on the optic nerve we are limited to a ∼3 mm diameter area of the posterior pole.

### Fluorescein retention and changes to angiography dynamics identified by pixel-by-pixel analysis

For validation, the analysis method was implemented in a cohort of animals that had undergone laser photocoagulation to cause disruption to the blood retinal barrier. Thirty minutes after the retina had been laser injured; fluorescein angiography was performed in 7 eyes. Although prolonged fluorescence can be detected from the fluorescein angiogram at a particular time point (Figure 6A), the size and severity of the abnormal fluorescence cannot easily be quantified. The pixel-by-pixel half-fall (Figure 6B) and offset (Figure 6C) analysis clearly highlights the size and extent of the four laser lesions compared to their surrounding capillary/background areas (the large retinal vessels and immediate surroundings have been masked). In these particular areas some pixels do not fall in fluorescence (red trace in Figure 6D), whereas others show an elevated offset (green trace in Figure 6D) when compared with the dynamics of a pixel from the surrounding retina (blue trace in Figure 6D).

This analysis method is able to highlight the diversity of fluorescein angiography dynamics in various areas of the retina. Figure 7 shows individual probability maps for half-fall (1^st^ and 3^rd^ rows) and offset (2^nd^ and 4^th^ rows) for each of the 7 eyes that had undergone laser injury. The half-fall maps help to differentiate those areas where fluorescence continues to increase or show minimal decay compared with the rest of the retina. On the other hand, the offset indicates all areas of the retina where there is excess fluorescence. These types of maps can be used to return global indices of abnormal fluorescence, to facilitate monitoring across longitudinal trials.

## Discussion

Fluorescein angiography is a useful tool for *in vivo* assessment of the blood-retinal-barrier both in clinic and laboratory experiments. Building on previous studies that have utilized dynamic analysis on contrast enhanced imaging [17], [23], [29], [31]; our pixel-by-pixel dynamic analysis method returns a suite of parameters that allows quantification of fluorescein dynamics across the entire image. This analysis method reliably delineates arteries and veins from the surrounding retina, determines the rate of fluorescence decay and locates areas of abnormality.

Laboratory studies largely deliver fluorescein via subcutaneous or intraperitoneal injection to identify blood-retinal-barrier compromise [13], [15], [32], [33]. Whilst this is an easy method of dye administration, it provides limited temporal information. Although intravenous fluorescein delivery is widely used clinically, as well as in some laboratories, its analysis is largely confined to a qualitative appraisal of areas of abnormal fluorescence (similar to our ‘offset’ parameter but without spatial quantification of the lesion). Regardless, a major problem with all methods of fluorescein delivery is that the extent of fluorescence is not static. Indeed, in areas of injury the fluorescence profile may not fit the canonical form (see Figure 6D). A good example of this problem is in the interpretation of the ‘offset’ in the laser injury, where pixels showing increased fluorescence could reflect injury at that site or could reflect the spreading of fluorescein from adjacent areas. Thus the size of the lesion is critically dependent on the timing of image acquisition. Without accurate timing, temporal changes in fluorescein can hamper quantification. Our analysis provides the half-fall to complement the offset, as it differentiates between those pixels that demonstrate a continuous increase in brightness, compared with those that show slow or minimal decay (compare upper and lower panels in Figure 7G). This may prove to be a more robust index for quantification of the spatial extent of injury in longitudinal studies or therapeutic interventions.

We have used the laser model here to demonstrate the utility of our analysis in terms of the derived half-fall and offset parameters. It is worth mentioning that our approach is also sensitive to small differences in the half-rise, particularly that between arteries, veins and capillaries. Previous studies have employed the arterio-venous transit time (or mean circulation time) as an index of blood flow velocity [28]. Our pixel-by-pixel approach can return information about local delays in circulation dynamics.

Previous studies have either manually segmented or employed an automated method to count microaneurysms in diabetic fluorescein angiograms as a means to monitor disease progression [34], [35]. Again care must be taken with such static quantitative approaches, given the temporal changes in the signal to noise ratio of fluorescein associated with the quenching of fluorescence, binding to albumin, dye dilution and leakage [36]. Our approach attempts to deal with these potential limitations by statistically segmenting pixels that show abnormal temporal characteristics. In this way it avoids confounds associated with differences in absolute fluorescein brightness and issues associated with the dimming of the image periphery as found with any optical imaging system.

The sensitivity of the approach to detect subtle changes relies on several experimental procedures that help to minimize sources of error. It is important that the delivery of fluorescein is standardized. In this study, a syringe pump was used to standardize the rate and volume of fluorescein delivery into the femoral vein. The size and length of cannula tubing was matched between animals to ensure that there was no difference in resistance. Furthermore, all lines were primed to have fluorescein 3 cm from the tip of the intravenous cannula prior to fluorescein infusion. Although at the time of experimentation the starting of the syringe pump and video recording could not be automated, the same operator was responsible for all recordings and thus inter-experimenter variability was kept to a minimum. In future, variability would decrease further with simultaneous triggering of the pump and image recording.

Blood pressure should be monitored during angiography as changes in this parameter can contribute to changes in circulation dynamics. Blood pressure was continuously monitored throughout this study via a femoral artery cannulation and returned stable recordings (mean blood pressure: 116.1±7.5 mmHg), which is similar to that previously reported for anaesthetized rats [37]–[39].

Image stability is also important to facilitate accurate pixel analysis. Whilst image registration and PCA helps to remove most eye movements and movement associated with breathing, the best approach is to ensure adequate depth of anesthesia. All animals in this study were measured at a similar time following the onset of anesthesia, approximately 1 hour after injection, due to surgery prior to imaging. Our preliminary experiments show that the current image registration and analysis approach works with 10–15 minutes of anesthesia induction (data not shown). Additionally, animals were lightly secured to the imaging platform via a neck strap, which helps to dampen breathing related movements. In this regard further improvement could be gained by using ear bars.

Despite these limitations, pilot data undertaken in 3 animals suggest that parameters returned from our analysis approach are reproducible. When fluorescein angiography is repeated 30 minutes apart, we find that half-rise (Arteries 0.02±0.03 s; veins 0.3±0.2 s, capillaries/choroid 0.2±0.2 s) and half-fall (Arteries 1.1±0.6 s; veins 0.7±0.4 s, capillaries/choroid 0.9±0.5 s), in the inferior quadrant inner eccentricity zone show very little difference.

Our study was acute in nature; however, the procedure can be adapted to longitudinal investigations. In particular, our temporary surgical cannulation of femoral vein can be replaced by chronic procedures (indwelling femoral cannulas for fluorescein injection and blood pressure monitoring) or less invasive approaches. For example, a tail vein injection allows for repeated fluorescein delivery along with non-invasive tail cuff sphygmomanometry.

## Conclusions

Pixel-by-pixel analysis of video fluorescein angiography has clear advantages over conventional, static angiography in allowing the quantification of fluorescence dynamics across the entire image and automated, objective identification of areas exhibiting abnormal fluorescein flow. This approach can be used for longitudinal studies to allow quantification of disease progression and treatment efficacy in vascular diseases such as diabetes, choroidal neovascularization and retinopathy of prematurity. The simplicity of the analysis means that it can be easily applied to video angiography acquired in clinical or laboratory settings using any number of contrast-enhancing dyes such as lucifer yellow and indocyanine green. Future studies may elucidate its value in human fluorescein angiography.

## Supporting Information

Video S1 **Video fluorescein angiography of the rat posterior retina.** Images were acquired at 30 Hz over 30 seconds.
(AVI)

File S1 **Matlab code to register a tiff stack.**
(M) Click here for additional data file.

File S2 **Matlab code to perform principle component analysis on a tiff stack.**
(M) Click here for additional data file.

File S3 **Matlab code to read in the trace masking the major blood vessels.**
(M) Click here for additional data file.

File S4 **Matlab code to fit the intensity profiles.**
(M) Click here for additional data file.

File S5 **Matlab code bin.m bins an image.**
(M) Click here for additional data file.

File S6 **Matlab code circle.m adds a circle to a plotted figure.**
(M) Click here for additional data file.

File S7 **Matlab code find_com.m finds teh centre of mass of a matrix.**
(M) Click here for additional data file.

File S8 **Matlab code fitThisPixel.m finds the intensity profile parameters.**
(M) Click here for additional data file.
